# System Mapping of Antimicrobial Resistance to Combat a Rising Global Health Crisis

**DOI:** 10.3389/fpubh.2022.816943

**Published:** 2022-06-17

**Authors:** Lea Ellen Matthiessen, Tine Hald, Håkan Vigre

**Affiliations:** National Food Institute, Technical University of Denmark, Lyngby, Denmark

**Keywords:** antimicrobial resistance (AMR), systems thinking, leverage points, system change, transformation, wicked problem, antimicrobial stewardship (AMS), sustainability transition

## Abstract

Antimicrobial resistance (AMR) decreases the effectiveness of antimicrobials to treat bacterial infections in humans and animals. The increased occurrence of AMR in bacterial population in humans, animals, and the environment requires the measures to combat a rising global health crisis. The aim of this research was to present current knowledge on AMR in a system map and to identify potential explanations of former identified variables significantly associated with AMR. This study applies a systems thinking approach and uses feedback loops to visualize the interconnections between human, animal, and environmental components in a circular AMR system map model. First, a literature review focusing on AMR and socioeconomic factors, wicked problem, and system change was carried out, which was then processed in a system map to conceptualize the present core challenges of AMR *via* feedback loops. Second, to investigate possible underlying values of the society and those that influence humans' behavior in the present AMR system, an iceberg model was established. Third, leverage points were assessed to estimate which kinds of interventions would have the greatest effect to mitigate AMR in the system. The present AMR system map implies the potential to identify and visualize important risk factors that are direct or indirect drivers of AMR. Our results show that the tool of system mapping, which interconnects animals, humans, and environment in one model, can approach AMR holistically and be used to assess potential powerful entry points for system wide interventions. This study shows that system maps are beneficial as a model to predict the relative effect of different interventions and adapt to rapidly changing environments in a complex world. Systems thinking is considered as a complementing approach to the statistical thinking, and further research is needed to evaluate the use of such tools for the development and monitoring of interventions.

## Introduction

Over the past decades, the rise of antimicrobial resistance (AMR) in humans, animals, and the environment represents a rising global health crisis (1). Consequently, due to the decreasing effectiveness of antimicrobials, it is predicted that AMR will be responsible for approximately 10 million death by 2050 (2). The cause of AMR is the selective pressure caused by antimicrobial usage (AMU), thereby favoring bacterial clones carrying AMR genes whereby they become the dominate bacteria causing infection. However, several determinants of health, including the lack of clean water, sanitation, and hygiene (WASH), poor hygiene management for disease prevention, as well as the lack of awareness and knowledge have a significant direct or indirect effect on AMR (1, 3, 4).

Traditionally, use, and especially large use, of antimicrobials was perceived as the determining factor causing AMR. This is reflected in several AMR research studies, particularly focusing on hospital settings (5, 6). However, other studies have recognized that health determinants, such as poverty, are also the important drivers of high levels of AMR (3, 4, 7, 8). Thus, mitigating AMR requires a systemic change to ensure sustainable development (5, 9). These sustainability transitions are acknowledged as “*(…) long-term, multi-dimensional, and fundamental transformation processes through which established socio-technical systems shift to more sustainable modes of production and consumption*” (10) (p. 956).

Over the last 2 centuries, the dominating scientific approaches relied on splitting complex situations into smaller pieces to understand them (11, 12). This “dissective” thinking has its advantages, such as making things measurable. Simultaneously, it involves a risk to dismiss the relationships between system components, which often define the functioning of a system. Therefore, the systems thinking approach according to Meadows (13) is considered as a helpful tool. On the one hand, to include the complexity of AMR and, on the other, to contribute to the establishment of powerful policy interventions. To the authors' knowledge, this is the first AMR system map, which is outlining AMR and its system elements with feedback loops. This holistic approach of assessing a problem from a systems' perspective can contribute to adding on knowledge to understand the complexity of AMR.

Systems thinking is an approach practiced in diverse scientific fields, such as social sciences, engineering, business and management, computer science, and medicine (12). However, there is not one consistent definition; it can rather be understood as a way of thinking and applying knowledge holistically. In global health, the advancements of systems thinking were outlined by Peters (14), who recognizes “*Systems thinking adds to the theories methods and tools we otherwise use in global health, and provides new opportunities to understand and continuously test and revise our understanding of the nature of things, including how to intervene to improve people's health*.” (14) (p. 5). Nevertheless, while systems thinking has been evident in the understanding of AMR as a systemic problem, it has not been explicitly used as a mapping tool to visualize potential leverage points (i.e., interventions that have the power to initiate system wide change).

System dynamics (i.e., feedback loops), an analysis on relationships among system components, can, on the one hand, demonstrate effective policies that must be maintained and, on the other hand, dismantle those that need to be reinvented (12, 15). Systems are continuously under development that influence all elements involved in the system (i.e., stakeholders and system dynamics) (16). This requires continuous evaluation, including the establishment and reconsiderations of new approaches, to solve complex problems rising from the system dynamics.

Antimicrobial resistance is a sustainability issue that can be described as a wicked problem (17–19). The effects of the wicked problem are severe and include potentially large adverse effects on the long-term, including social, economic, and environmental pillars. Typically, these problems include larger time lags from the cause to the effect and include an extensive number of actors involved (20). In terms of AMR, there is a time lag between the application of antimicrobials and the spread of multi- and pan-resistant bacteria (1, 7). On the one hand, the application (i.e., AMU) of antibiotics depends on the local circumstances, such as health coverage, accessibility, and treatment costs (21). Additionally, multiple risk factors influence the development of AMR. Important risk factors include previous antibiotic exposure, underlying disease, and invasive procedures (5, 7). On the other hand, different drivers facilitate the spread of resistant bacteria, such as animal and water sources (22). Globally, the current spread seems to occur rapidly (1, 23). Moreover, the AMR network involves a large number of stakeholders. For instance, the micro-level includes patients and their relatives (1); the meso-level cover the medical representatives, pharmaceutical companies, animal and agricultural industry, and research institutes (2). Finally, the overarching macro-scale incorporates national and international regulators and policymakers (3) (24).

The diversity and abundance of AMR vary across the world. A global study that investigates the occurrence of AMR determinants in sewage samples indicates that AMR abundance in most geographical parts is influenced by local or national drivers (4). Furthermore, Henriksen et al. (2019) suggested that advancements of sanitation, health, and education have a potential to decrease AMR. There are two overall aims of the work presented in this paper: first, to outline a system map of the present AMR system taking into account current knowledge on AMR, second, based on the papers reviewed to elaborate on potential pathways, how improvements of sanitation, health, and knowledge, influence the AMR system, expressed as feedback loops. This approach was applied to identify potential leverage points striving toward sustainable development (25, 26). Our approach combines holistic systems thinking with previous knowledge gained from linear epidemiological models.

## Methods and Materials

Based on collective knowledge, this study approaches AMR through systems thinking. For our study, this implies that different elements directly or indirectly influencing AMR in the human, animal, and environmental setting are set into the perspective of each other. This qualitative research design leads to a systems' view that can be plotted into a system map (**Figure 3**).

**Figure 1:**
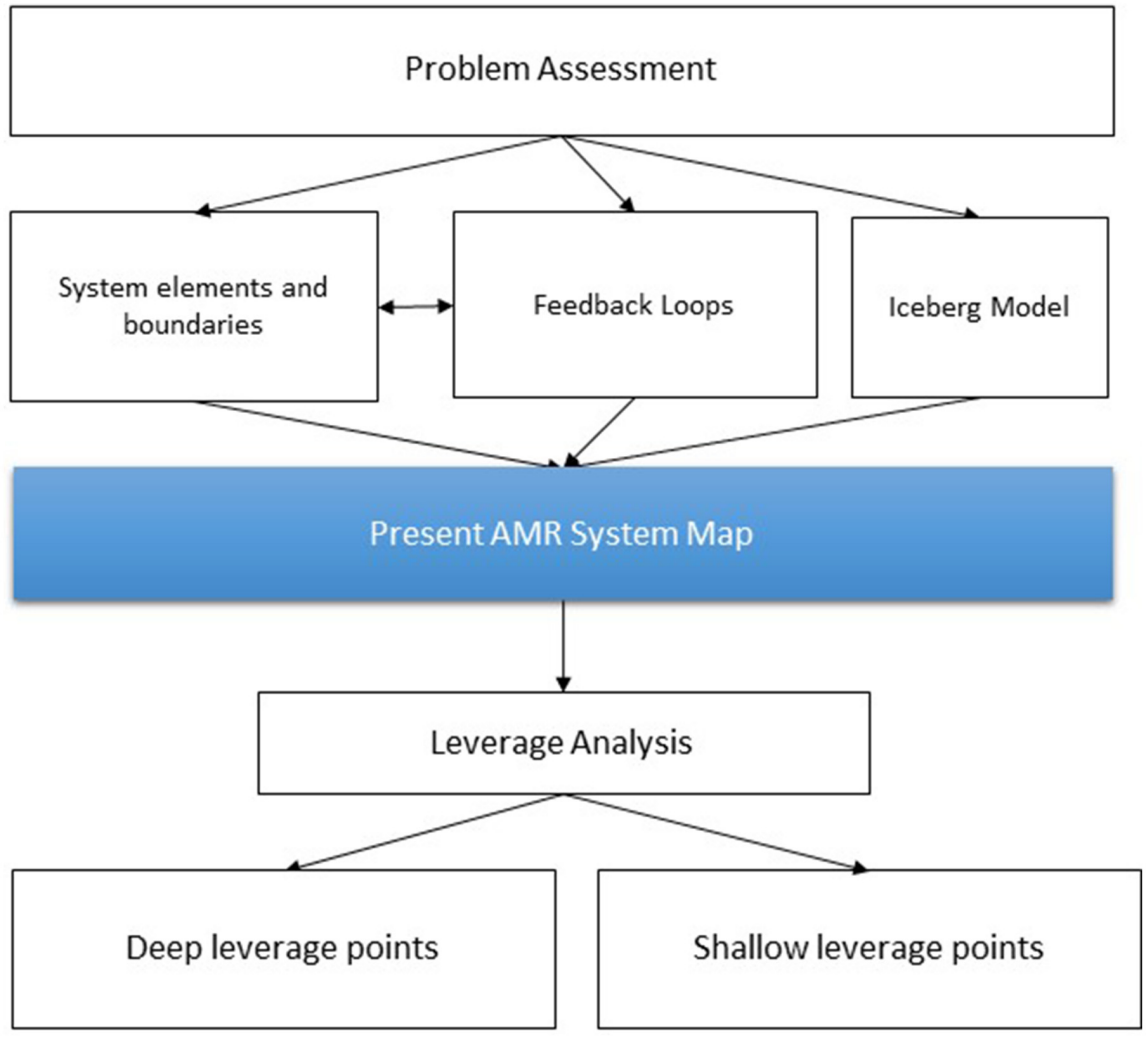
Flowchart of study design using a systems thinking approach. In terms of the system mapping and identification of leverage points following Meadows (13) and Abson et al. (11) and in terms of the Iceberg Model following Monat and Gannon (12).

Figure 1 displays the study design, which is based on sustainability science applying a systems thinking approach (11–13). An overview of the systems thinking terminology used here can be found in **Supplementary Table S1**.

First, the problem of AMR was assessed based on the literature research performed in August to September 2020. Publications not older than 5 years were selected (2015–2020). Table 1 depicts an overview of a series of keywords searched for in two different databases. The search strings of the literature review did not focus on the individual level but rather on socioeconomic factors potentially correlating with AMR (4, 22) (Supplementary Table S2). For each series of keywords, the first ten results were considered only. First title was skimmed to exclude papers not relating to AMR. For instance, papers relating to stakeholder analysis and sustainable management approaches were excluded. Then, abstract was reviewed to identify the common drivers of AMR. Risk factors on an individual level were not considered why studies mainly in a clinical setting were excluded. Studies about antimicrobials on an individual level, such as studies in clinical settings, were excluded.

**Table 1:** Literature research to identify the drivers that influence antimicrobial resistance (AMR).

**Database**	**Search string**
Scopus	amr AND global AND surveillance
	amr AND “global surveillance”
	amr AND “socio- economic factor”
	amr AND “educational attainment”
	amr AND “infection and malnutrition”
	amr AND “cultural Traditions”
	amr AND review
	amr AND review AND intervention
	amr AND review AND policy
	amr AND review AND wicked problem
	amr AND “one health” AND agriculture
Google Scholar	amr AND “socio-economic factor”
	amr AND “one health”
	antimicrobial resistance surveillance one health
	antimicrobial resistance AND “one health” AND gene
	amr AND review AND “wicked problem”

*The table depicts an overview of the search items (second column). The timeline of research was August to September 2020. In addition, a filter was applied, not older than 5 years (2020–2015), to limit the results for publications, and only the first 10 results were viewed*.

Second, all potential system elements relating to the AMR system were compiled, and boundaries of the AMR system were set based on the literature research and the feasibility to make policy interventions. Next, these system elements were interlinked using positive and negative feedback loops. Third, the AMR system was assessed based on the Iceberg Model. Fourth, to identify potential levers for interventions, a leverage analysis of the present AMR system map was conducted, identifying deep and shallow leverage points.

### Defining System Elements and Boundaries

“*A system is an interconnected set of elements that is coherently organized in a way that achieves something*” (13) (p. 11). To study systems, a system can be characterized by three objects: elements, interconnections, and a function or purpose. The present AMR system was defined, including the purpose, the system elements, and setting the system boundaries (13). In relation to the scope of this research, system elements were chosen to identify the root of the problem and based on the ability to intervene on. Overall, it needs to be emphasized that the system elements that are displayed in the present AMR system map were highlighted (e.g., “name of system element”) for improved clarification.

The list of elements in a system can be extended indefinitely; therefore, it is most important to look at the interconnections that are a key part of the system. These interconnections are expressed as feedback loops (13). Thus, system elements were assessed as emergent properties that are defined by its interconnections rather than individually (12). Socioeconomic factors reported by the World Bank associated with AMR abundance were considered in the system elements (4). For instance, “affluence” represents welfare [e.g., Human Development Index (HDI)], “infection and malnutrition,” and “available resources” (e.g., open defecation practice). In addition, “available resources” include access to clean water and sanitation (i.e., WASH). In the next step, the system boundaries were defined. The AMR system is affected by changes in the interconnecting systems, and reversely, the AMR system affects the interconnecting systems.

### Feedback Loops

Next, the system elements were interconnected, and causal loops were assessed with positive and negative feedback loops (13, 15). The knowledge depicted on the AMR system maps is based on the literature research and further discussed by diverse scholars in an online workshop that work in the fields of AMR and One Health. Such system dynamics try to give a more realistic perspective to the modeling and depict interconnections that stabilize (negative feedback loop) or destabilize (positive feedback loop) a system framed by feedback mechanisms (12). Negative feedback loops are in charge to regulate the system in a self-regulating manner, under different conditions and impacts. Positive feedback loops are self-reinforcing. These cause growth, explosion, erosion, and collapse in systems. Never-ending positive feedback loops would destroy a system (“racing to the bottom”) (13). Instead of linear cause–effect relationships, systems thinking depicts the dynamics as causal loops (circular) (15).

The present AMR system map was generated as an iterative process. First, the system mapping was applied manually by the use of post-its to transfer the knowledge of the current state of AMR from the literature review to a system map. Next, the handcrafted system map was transferred to an online tool. For the layout, a simple online program, “Kumu,” was used including an animation function of the feedback loops (27). Different scientists discussed and further established the interconnections between different system elements. However, the AMR system map must be acknowledged as a tool that can be further developed continuously.

The “state of the system” is the stock, which for the present AMR system map are the overall bacteria population in the system (13). This means, if AMU is increased, the number of resistant bacteria will increase. However, for the flows to accumulate and disseminate in a stock will take time, which is symbolized with a time delay on the present AMR system map (1). This delay is caused by the conditions of microbes and circumstances of the individual situation (i.e., previous antibiotic exposure, underlying disease, and exposure at work) as well as WASH parameters (1, 7). Not all interconnections are physical flows (i.e., hard variables). According to Meadows (13), flows between system elements also entail information, such as the access to education (i.e., soft variables).

In this work, the flows aim to describe potential scenarios as researched by the literature (**Supplementary Table S3**) and are furthermore based on some assumptions made by the authors. Assumptions were applied to enable establishing an overview of the AMR situation, predominantly described by the literature, without involvement in local variances. For example, that the larger part of agriculture is based on intensified animal production with an interdependency on AMU. This is an assumption because it simplifies the general situation. First, intensified animal production can also be managed without AMU with an adequate hygiene management. Second, the extent of intensified agriculture varies across countries. In developed countries, AMU is widely embedded into the food system (28, 29). Additionally, AMU may both contain a cultural perspective and is a matter of resources (i.e., accessibility). Third, in most developed countries, people can go to a doctor whenever they like. In contrast, in Africa, there may be regions, especially in rural abundant places, where access to the health system is not assured (21, 30).

System elements and boundaries, together with the system dynamics (i.e., feedback loops), facilitated the system mapping.

### Iceberg Model

**Figure 2:**
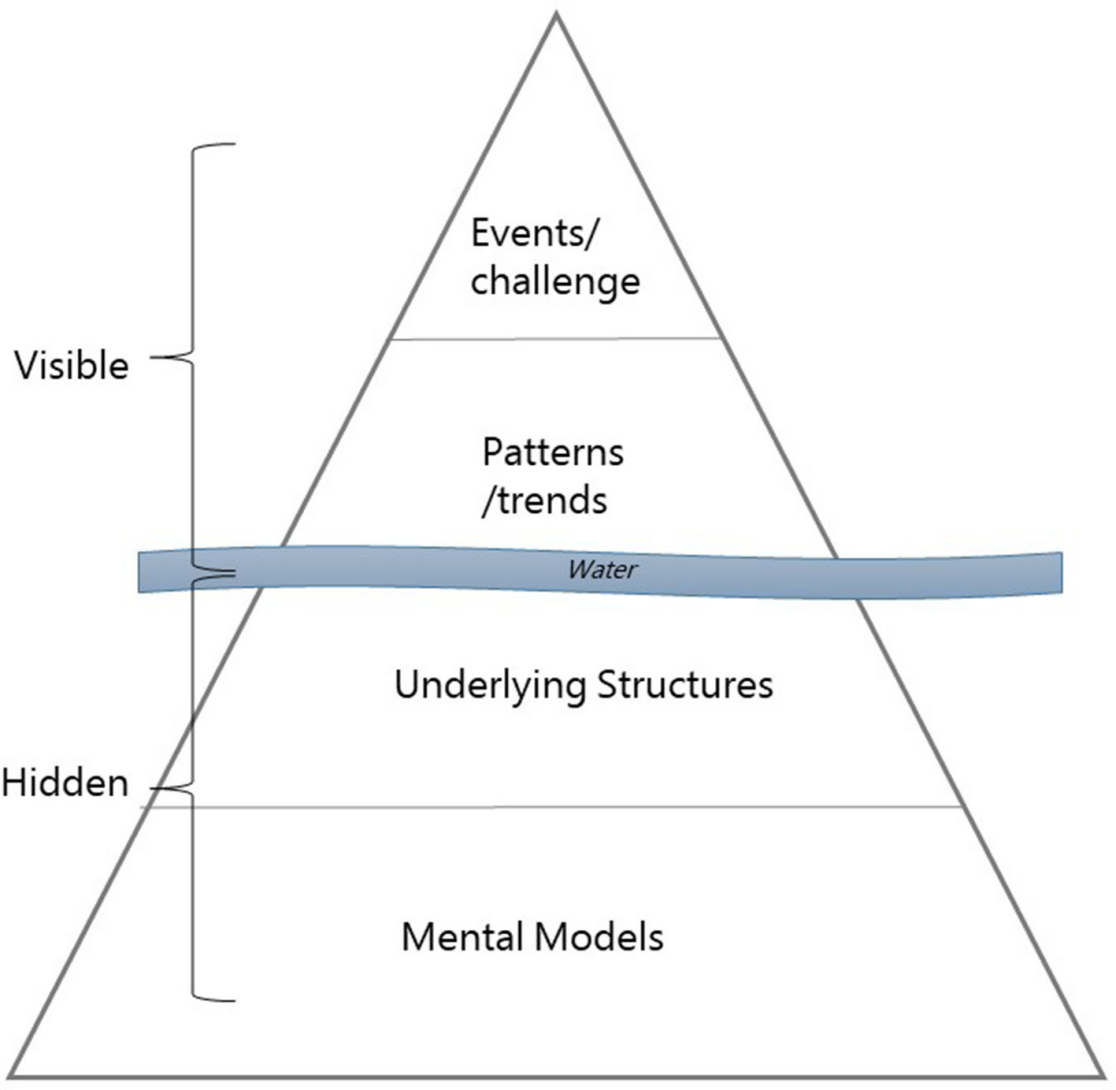
The iceberg model of a human-designed system. The model consists of two visible components above the water surface and two hidden components beneath the water surface. Repeated events demolish the patterns of the system, which are influenced by system structures. Mental models are people's behavior that is shaped by the system structures. Drawing inspired by (12).

The iceberg model is a crucial component of systems thinking because it uncovers mental models, which can be understood as the underlying values of a society and those that influence humans' behavior. The iceberg model is a way to think deeper of a problem, and it allows seeing different layers of a system, such as mapping patterns and trends or underlying belief structures. In systems that are herded by humans, the system structure develops as a consequence of mental models or paradigms (12). Figure 2 shows the iceberg model, which consists of a visible and a hidden part. The concept of the iceberg model anticipates that repeated situations of a system dismantle patterns (12). The events are on the top. Events are situations that we meet on a day-to-day basis. In general, events are recognized more easily than noticing patterns. Patterns are the accumulated memories of such events. Events and patterns are the mechanisms through which mental models get adapted into action (15). These are essentially caused by the structure of the system (12, 15). In this study, we answered the questions listed in **Figure 5** based on the literature research. The questions were developed as a part of the system mapping.

### Leverage Point Perspective

System leverage points tries to find answers on where in a system, we should intervene to change its overall behavior. Within a system, it is important to find the area, where an intervention would have the greatest effect. Meadows (13) introduced 12 *Places to Intervene in a System* as a hierarchy of intervention points, which was used to assess the present AMR system (i.e., leverage analysis). Meadow's hierarchy model (i.e., intent, design, feedbacks, and parameters) was applied to the findings on AMR-related issues in the literature (**Supplementary Table S4**). Different interventions that intent to mitigate AMR are listed and then categorized: First, deep leverage points have a more deeply embedded effect on the system. Second, shallow leverage points influence the system to a smaller extent (11, 25, 26). Deep leverage points are “*Places in complex systems where a small shift may lead to fundamental changes in the system as a whole*.” (11). In terms of AMR, it is anticipated that a deep leverage point does not encompass AMU but is rather deeply embedded societal norms and values.

## Results

According to several scholars (2, 3, 7, 28, 31) and international organizations (1, 23, 32), AMR may hamper the treatment of bacterial infections, whereas the resistance bacteria or genes are spread naturally between humans, animals, and the environment. Based on this, the function of the AMR system was determined *to hinder the exposure to bacteria with resistance genes in order to maintain the effectiveness of antimicrobials*.

### Present AMR System Map

**Figure 3:**
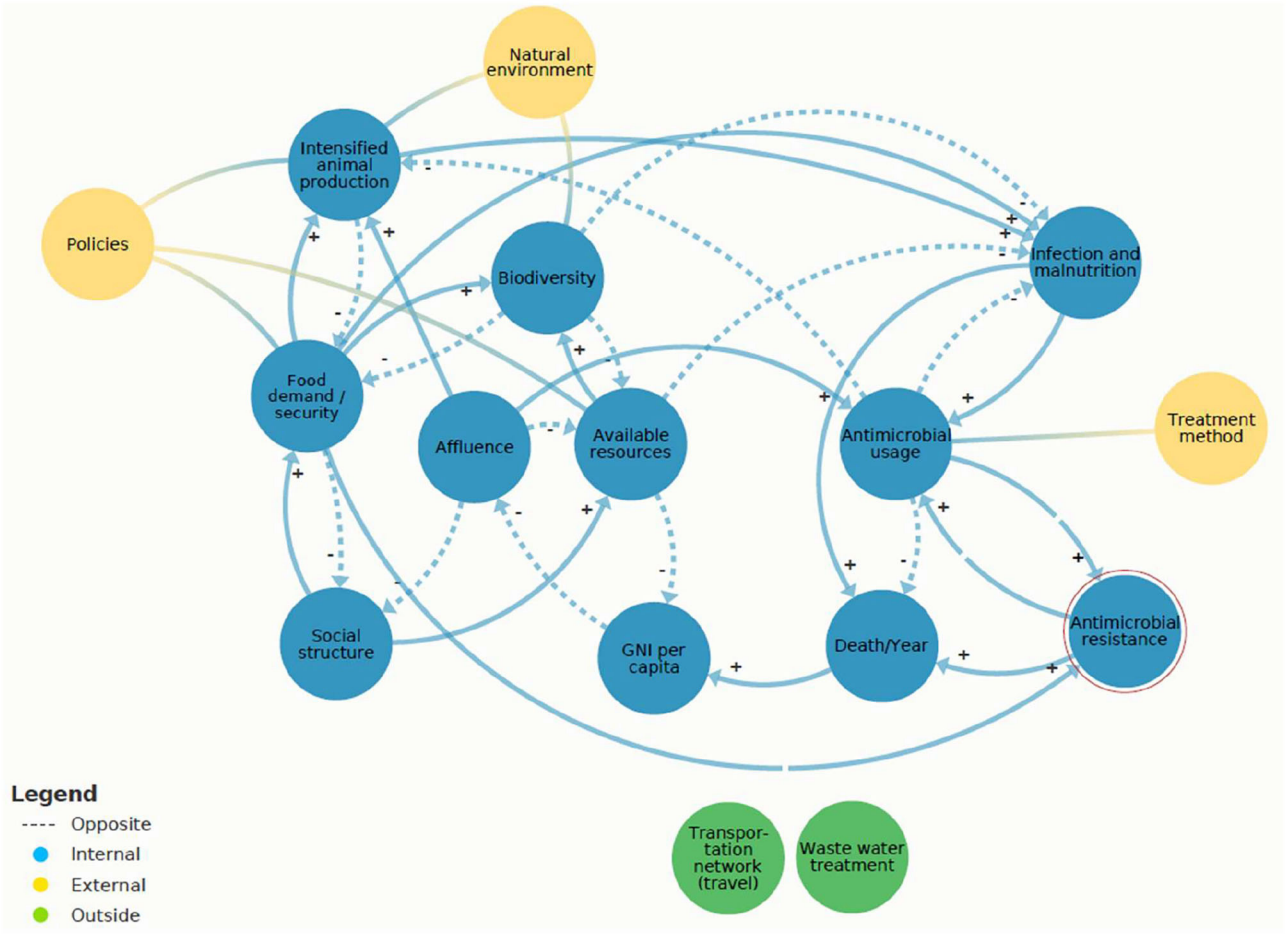
Present antimicrobial resistance (AMR) system map created in Kumu.io (27) https://www.kumu.io/LeaMat20/amr-system-map#untitled-map. The internal system elements (blue) are connected with arrows indicating the negative (balancing) and the positive (reinforcing) feedback loops. These interconnections are based on the hard variables (i.e., physical flows) and soft variables (i.e., information flows). The breaks in the arrows, which are leading to “AMR,” indicate a time delay. The three yellow circles (i.e., policies, natural environment, and treatment method) depict the external elements that affect the internal objects. The two green elements (i.e., transportation network and wastewater treatment) symbolize systems outside of the system boundaries.

Figure 3 displays the present AMR system map. The model contains 17 system elements, 11 of them refer to the internal AMR system (blue). The latter are the core of the analysis. Moreover, important elements, externally connected to the main elements, were “policies” (e.g., legislations and surveillance), “natural environment” (e.g., spread of antimicrobial agents and depletion of resources), and “treatment methods” (e.g., education of medical staff and accessibility to medicine). The present AMR system map exemplifies two systems (i.e., “transportation network” and “wastewater treatment”) outside of the system boundaries. The economic system was partly integrated by the system elements “affluence” and Gross National Income (GNI) “GNI per capita,” and the medical system through “treatment method.” This model of the present AMR system map can be used to identify feedback loops and its effects on AMR.

Main elements of the AMR system (Figure 3) were “available resources” (e.g., access to health, sanitation, and knowledge) and “AMU”. “AMU” was reinforced by “infection and malnutrition,” including the origin of animals due to “intensified animal production.” On the present AMR system map, countries were represented as “social structure.”

An overview of the system dynamics that interconnect the system elements in the following: to begin with, the society of the “social structure” demands food to survive and therefore reinforces the “food demand” (positive feedback loop). When the “food demand” is accomplished, it stabilizes the “social structure” (negative feedback loop). A total of two scenarios were identified that hinder the stabilizing negative feedback loop from “food demand” back to “social structure.” First, if the food supply is defected, for instance, due to unequal distribution of food in the society. Consequently, the “food security” would reinforce malnutrition (“infection and malnutrition”). Second, if the food supply is based on an unhealthy diet the “food demand” will also reinforce “malnutrition” (positive feedback loop) (33). Furthermore, the “food demand” was linked to “biodiversity.” Human population is growing worldwide (34), the “food demand” can stress “biodiversity” due to increasing land use (28, 35). Reversely, “biodiversity” can balance the food system through enhanced resilience (36, 37). Furthermore, “social structure” depends on “available resources” (e.g., WASH and knowledge) and therefore reinforces “available resources” (positive feedback loop). The “social structure” is balanced (negative feedback loop) by “affluence” (e.g., HDI). In sum, “social structure” was interrelated to “food demand and security,” “available resources,” and “affluence.” Depending on the circumstances of the “social structure,” the resistance organisms were likely to be enhanced or not. This is reflected by the finding that AMR abundance differs among different geographical locations (4).

**Figure 4:**
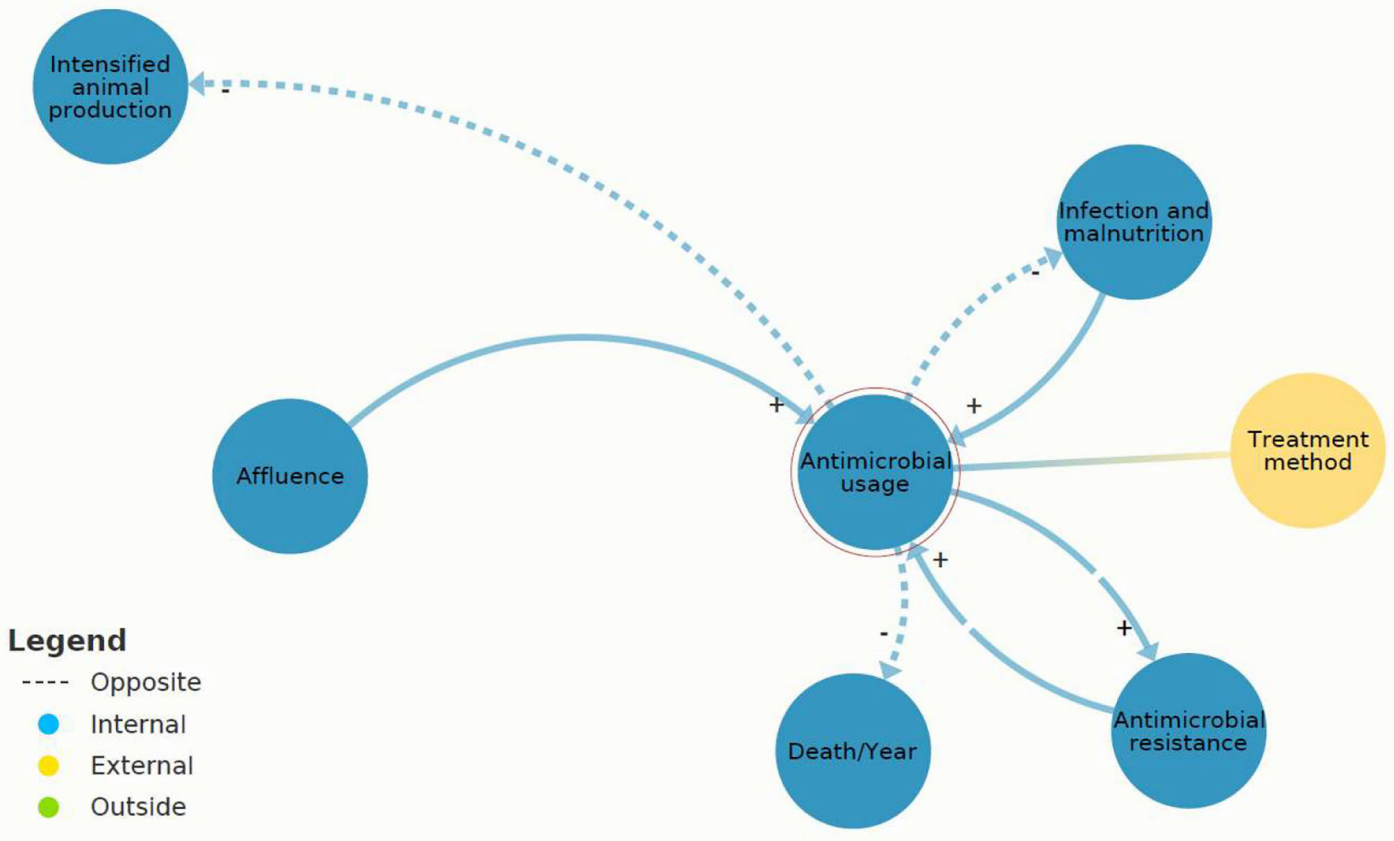
A section of Figure 3: causal loops in present antimicrobial resistance (AMR) system map between “antimicrobial usage” (AMU) and “AMR” created in Kumu.io (27).

Figure 4 shows the core cause of the AMR challenge, which was identified as a mutually reinforcing circle between “AMU” and “AMR.” The “AMU” reinforces “AMR” (positive feedback loop) because higher antibiotic usage increases the abundance of AMR.

### AMR System's Mental Model

**Figure 5:**
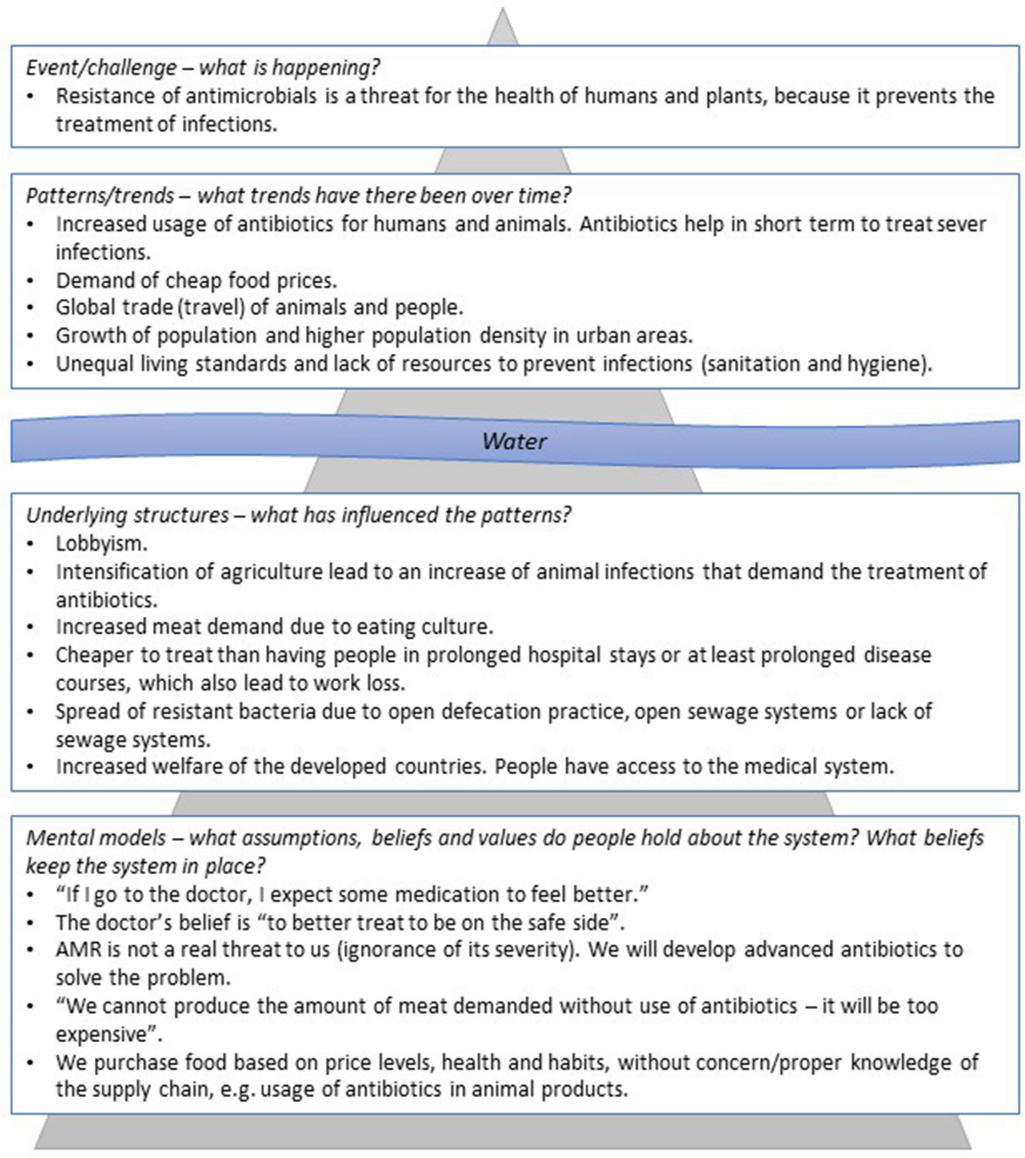
Iceberg model of present AMR system. On the top, visibly, the events and patterns of the present AMR system. Under the water surface, hidden, the system structure and on the bottom peoples' underlying assumptions, beliefs, and values (mental model). The upper part is supposed to be influenced by the one below, meaning the system structure is caused by the mental models. The model is applied lightly in this context to give an overview of the different levels and to provide a contextual application of the system map around the “water level.” To truly see the depth of the AMR problem, the iceberg model could be explored much more deeply to enter into the mental models that undercut all of society as we know it (e.g., life and death). However, this becomes a philosophical exercise and is outside of the focus of this research.

Figure 5 symbolizes the AMR system's assumptions, beliefs, and values. The results of the iceberg model *(*Figure 5) assume that antibiotics are perceived as an adequate treatment method among individuals (5). People seem not to be aware of its risks or do not care. Additionally, the belief in technology seems deeply embedded in the AMR system, because the research and development to discover new drugs is financially promoted (9).

### Leverage Points of AMR System Map

**Figure 6:**
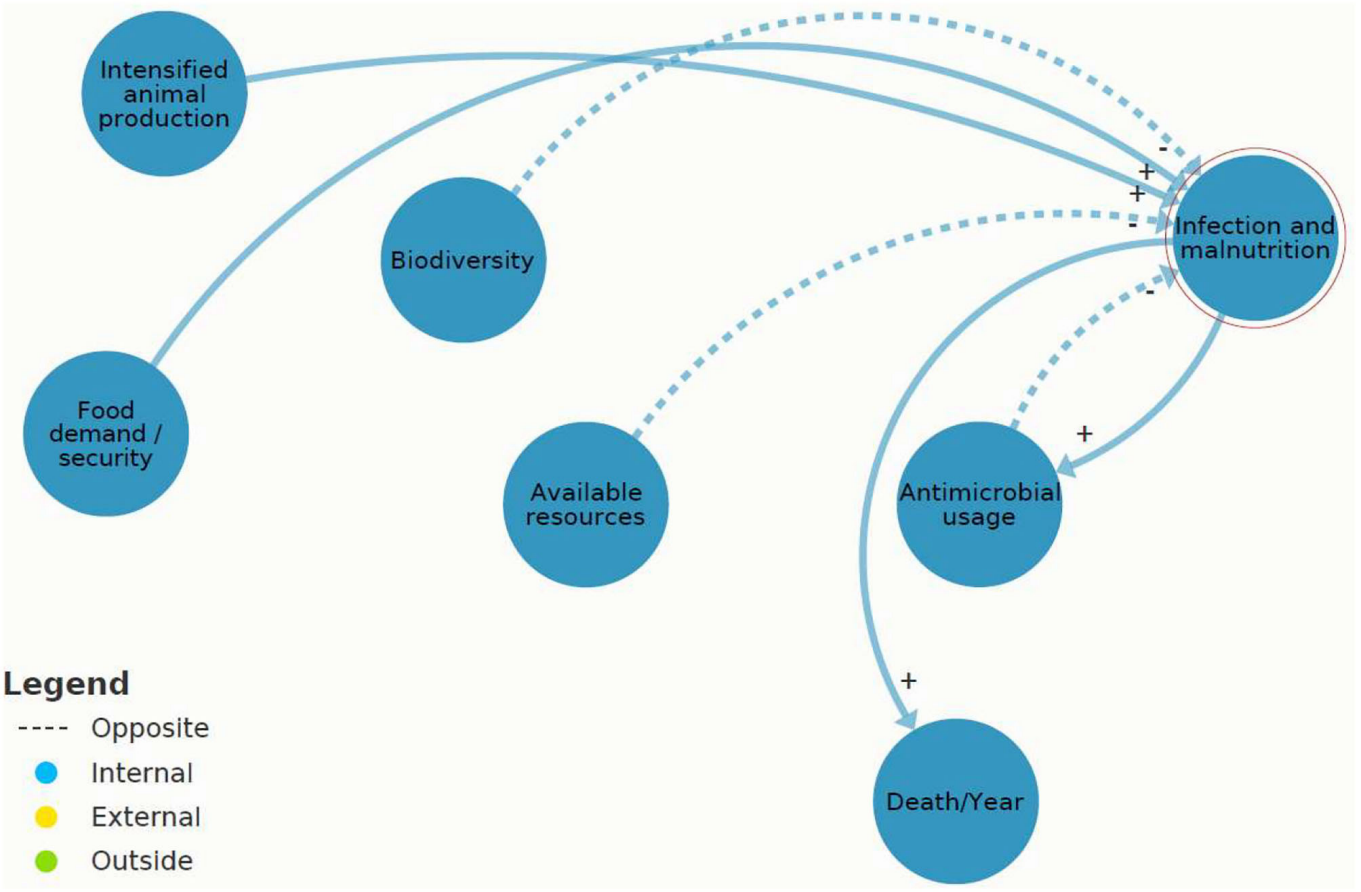
A section of Figure 3: causal loops in present antimicrobial resistance (AMR) map of “infection and malnutrition” (IM) created (2021) in Kumu.io.

**Figure 7:**
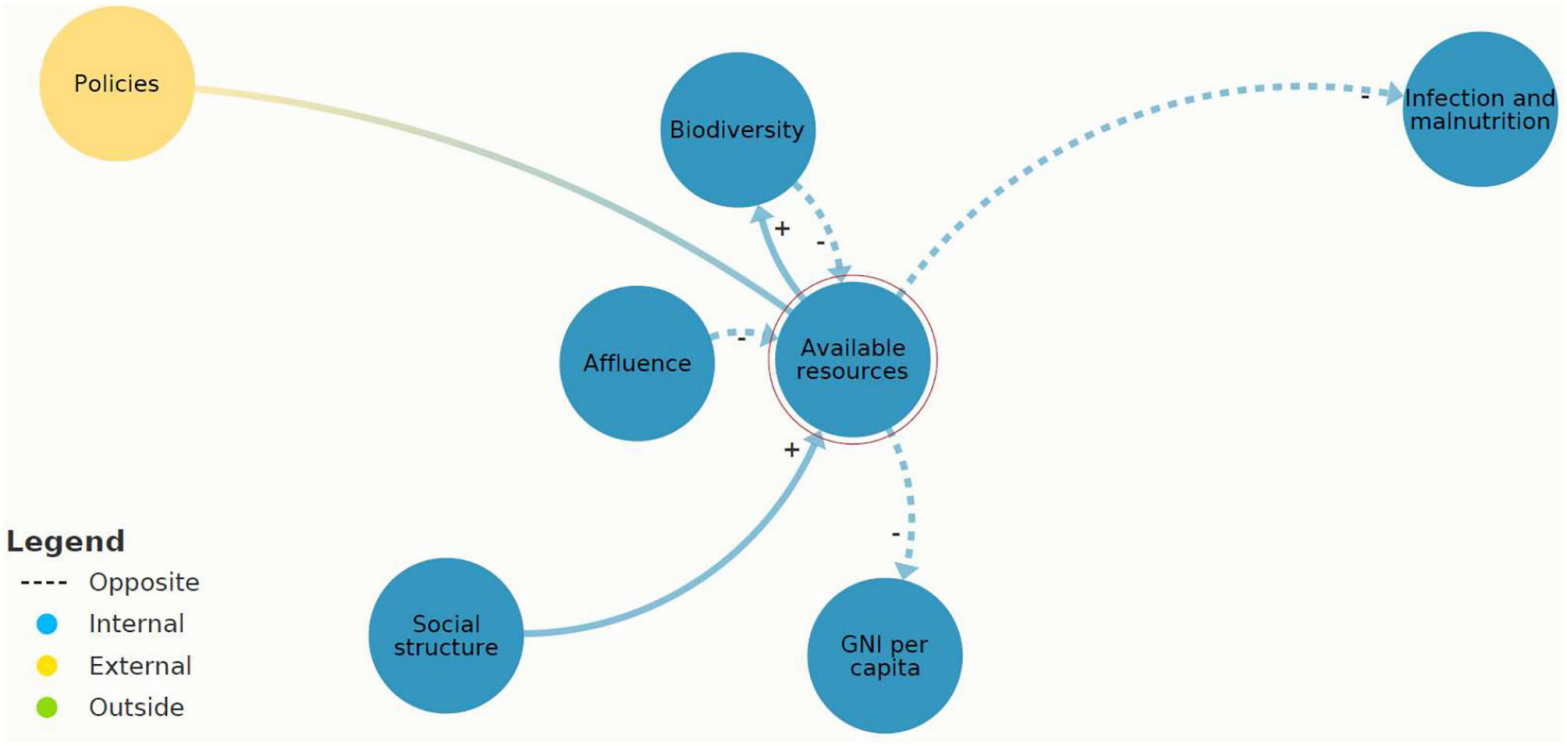
A section of Figure 3: causal loops in present antimicrobial resistance (AMR) system map of “available resources” created in Kumu.io (27).

Referring to the present AMR system map (Figure 3), three system elements showed leverage potential for system-wide change: “infection and malnutrition,” “intensified animal production,” and “available resources.” These leverage points are different in their effectiveness according to the leverage point hierarchy. “Infection and malnutrition” seem less efficient and “available resources” most efficient. The comprehensive analysis of the shallow and deep leverage points is available in Supplementary Table S4. For a simplified visualization of the complex dynamics, the two scenarios “infection and malnutrition” and “available resources” were depicted on “zoomed in” scenarios of the present AMR system map (Figures 6, 7).

Figure 6 indicates that “infection and malnutrition” affects “AMR” with one system element in between “AMU,” which reinforces “AMR.” A total of two flows put pressure on “infection and malnutrition,” and these were positively reinforcing loops (i.e., “food demand” and “intensified animal production”), which means an increase of “infection and malnutrition.” “Infection and malnutrition” was assessed as shallow leverage point, directly leading to (reinforcing) “AMU” or “death.”

Figure 7 demonstrates that the causal loops originating from “available resources” have the potential to balance “infection and malnutrition.” This system characteristic was assessed as a deep leverage point.

In sum, the most threatening feedback loop was found between “AMU” and “AMR.” “AMU” was reinforced by different causal loops, for instance, “intensified animal production.” In case, “AMU” was not effective or available, and it resulted in “death.” “Available resources” were identified as having a balancing effect on “infection and malnutrition” and can be recognized as a deep leverage point.

## Discussion

This study applied parts of the current scientific knowledge of AMR to a system approach. The literature research revealed AMR as a wicked problem (i.e., complex stakeholder network, spread of antimicrobial resistance genes between humans, animals, and the environment, and unforeseeable delay of the widespread consequences of AMR) (31, 38). This means that the aggregation of many factors leads to an increase of AMR. The tool of system mapping, which interconnects animals, humans, and environment in one model, can approach AMR holistically and contribute to apply the concept of “One Health.” The approach is in principal applicable in all countries, but the usefulness of identifying interventions will depend on how the regulatory system is organized in the country or in a regional context. To be efficient, the region in question should include a collaboration between all AMR stakeholders (i.e., public health, animal health, and environmental health). One Health has recognized AMR as the most complex global health threat, needing a multidisciplinary mitigation strategy (16, 22, 31).

As a result, five interconnected systems of the core AMR system were initially identified (i.e., economic, educational, health, transportation, and wastewater treatment systems), from which three were integrated into the present AMR system map model (i.e., economic, education, and health system). For instance, the economic system is linked to AMR due to the losses of productivity if people turn ill or die (i.e., “GNI per capita”). In addition, if pathogenic bacteria are resistant toward certain types of antibiotics, it requires more advanced treatment, which is more costly (21). Furthermore, the educational system influences people's knowledge and awareness, example wise on hygiene (1). The medical system assures optimal treatment of bacterial infections with antibiotics, but also in many case treatment without use of antibiotics (i.e., “treatment methods”), for instance, the use of vaccines. Both the medical and the educational systems were connected to the system elements “available resources” and “affluence” (i.e., HDI) (21). Outside the boundaries were the transportation and wastewater treatment systems, which are interrelated to the AMR system due to the spread of microbes, including antimicrobial resistance organisms, for instance, through trade, travel, and public transport (3, 8, 21).

Given the current scientific knowledge, and putting the information into system maps, we have shown that the present AMR system map require deep societal changes to mitigate future consequences of AMR. Therefore, it is important to recognize root causes, such as system structures, values, and goals, and understand the dynamics between system elements. Results from the iceberg model indicated that the mindset out of which the present AMR system arises underestimates the risk and consequences of AMU. This can be ascribed to the fact that outside of the hospital settings, it was estimated that approximately 30% of antibiotics would have been avoidable (5). Jørgensen et al. (9) recommend a social-ecological transformation to ensure a long-term sustainable use of antibiotics. Furthermore, they emphasize a bottom-up approach that includes collective consumer action (9). Therefore, individuals should be better informed about AMU and AMR. Thus, the antimicrobial stewardship (AMS) may be an adequate resource to decrease inappropriate AMU (5).

The present AMR system map showed that system elements “AMU” and “AMR” are mutually reinforcing each other (positive feedback loop), and this vicious circle must be interrupted. The virtuous circle can be explained by the following: if antibiotics become less effective, alternative antibiotics are applied, and therefore, the “AMR” reinforces “AMU” (21, 23). Alternative antibiotics may in turn be more expensive and/or give rise to more severe adverse effects (39–41). Consequently, these endless positive feedback loops stress the AMR system that can lead to a system collapse. However, “AMU” is difficult to intervene due the risk of prolonged illness or “death.” Instead, the system element “available resources” included deep leverage points that may have the potential to initiate sustainability transitions. Thus, system mapping has the potential to identify and visualize important risk factors that are indirect drivers of “AMU” and “AMR.”

Interventions strive to support the system's purpose, which is *to hinder the exposure to bacteria with resistance genes in order to maintain the effectiveness of antimicrobials*. Deep and shallow leverage points were assessed in the present AMR system (11). This method is useful to advocate for potential powerful interventions that aim toward system change and sustainability transitions (13). The mitigation of AMR includes, on the one hand, government policies (top-down approach), such as new rules for the system, and on the other hand, individuals that need to be empowered to strengthen their self-sufficiency (bottom-up approach) (11, 13). Several scholars advocate a social-ecological transformation to ensure a long-term sustainable use of antibiotics (5, 9).

According to the results, this means to avoid that the system will continue to have an increased “AMU,” which connects to an unpredictable rise of AMR and its consequences. “Available resources” were assessed as deep leverage points (i.e., intent and design) (11), which include hard and soft variables. First, there were the physical variables to ensure various hygienic conditions, such as access to sanitation facilities, clean water, and access to knowledge and the health system. Second, information flows could contribute to two major transformational steps: on the one hand, to raise awareness of risks on AMU, and on the other hand, to enhance knowledge on hygiene precautions to avoid “infection and malnutrition” of humans and animals (i.e., AMS) (1). Leverage points acknowledge the architecture's limitations and bottlenecks. Therefore, strong leverage points encourage self-organization to increase system resilience (13).

Abson et al. (11) found that a variety of sustainable interventions are targeted by highly tangible, but basically weak leverage points. These shallow leverage points do not indicate a great potential for transformational change. In the present AMR system map, “infection and malnutrition” was assessed as a shallow leverage point (i.e., parameters) (11). Typically, “infection and malnutrition” is treated with antibiotics, if applicable. However, “AMU” reinforces “AMR.” Further, “AMU” can cause diseases such as diarrhea due to a disruption of the normal gut microflora (42). Today's scientific problem framing of AMR gives the impression of mainly focusing on health. Maybe that is one of the reasons, why AMR has been assessed largely in hospital settings (6). There is a need to draw attention to the problem framing of AMR as a wicked problem embedded in the social, environmental, and economic scope (31).

Our model takes into consideration that impactful interventions include the provision of tools that grant knowledge and awareness. Improving on these system dynamics would contribute to ensure a long-term handling of AMU and meet the UN Sustainable Development Goals (SDG) [i.e., SDG 3 (Good health and well-being), and SDG 6 (clean water and sanitation)] (43). Starting with AMR's root causes a future AMR system map is supposed to target the following:

1. Supplies of resources (i.e., WASH), to strengthen the balancing feedback loops on “infection and malnutrition” as one of the main drivers of “AMU.”
2. Empowerment of livestock production without “AMU” as a precaution, to prevent reinforcing feedback loops by antibiotics.
3. Enhancing knowledge exchange (i.e., AMS) as an integrated part of the system to reinforce the spread of information that enables the handling of antimicrobials as a long-term approach on all system levels.

Sustainable transitions of the present AMR system map include different system levels (e.g., the macro-, meso-, and micro-level) (21). For instance, it needs the allocation of financial resources to fulfill WASH for all (1) and govern AMU in agriculture by regulation through an integrated pest management (32) or rather grant (existing) agriculture practice without AMU (2). On the meso-level, the community of smallholder farmers is likely to be empowered through growth in cooperation. Finally, the individual level citizens (3) are empowered to take a conscious choice. These interventions on AMR to handle antimicrobials as a long-term approach target a top-down and bottom-up approach.

This study has several strengths and weaknesses. The system approach has several strengths (26). First, due to the complexity of AMR, feedback loops can be seen as a relevant tool to visualize the dynamics between system elements. In addition, a leverage points perspective can contribute to conceptualize interactions among various interventions (26). Here, the iceberg model enables to see different layers of the system. Second, the use of systems thinking can be incorporated as a complementing approach to the traditional linear scientific approaches. If future research builds on this approach, it has the potential to give an improved understanding of reality and change the mindset of rather typical scientists in linear thinking. Third, feedback loops also depict soft variables, such as communication. *Fourth*, instead of linear cause–effect relationships, systems thinking depicts the dynamics as causal loops (circular) (15). According to Meadows (13) “the state of the system” is the stock that can be of physical or non-material origin. In- and outflows determine whether the state is balanced or reinforced. Negative or balancing feedback loops control the flows to bring the state to the desired level.

The method also has several limitations. First, feedback loops do not represent quantitative knowledge about the size of the effect. For instance, it is not clear to what extent hygiene prevention would have an effect on AMU. This holds true for all the arrows. Second, the authors build the arrows on some assumptions that do not cover regional and cultural differences accordingly. Articles that are sorted out may contain potential drivers of AMR, and there are other drivers that we do not know about. Identified drivers from high-income countries remain overrepresented. Thus, there is a lack of data for middle- and low-income countries. Furthermore, the content of the arrows build on layers identified in the iceberg model. This work focus was mainly on mapping patterns and trends to enable policy interventions. However, the mapping could also have chosen a focus on mental models (i.e., belief structures). Third, many of the systems thinking tools could be interpreted differently, because the interpretation can include a personal bias (12). For instance, the iceberg model is based on the less evidence and the content arbitrary. On the one hand, the evidence could be enhanced through further research, for instance, focus group interviews. On the other hand, the mental models could have been drawn more attention as systemic change means assessing core values of a society. This would imply to acknowledge the base model of consumerism (i.e., non-transparent supply chain, such as hiding usage of antibiotics in food production) or human's attitude to life and death (i.e., the circle of life is not related to acceptance of death but rather expectations of healing and a long-lasting life). This more philosophic thinking would need further research. Fourth, delays of feedback loops are frequently causing oscillations. The intend to regulate the system to a goal cannot be controlled reliably. By receiving delayed information about what the state of the system is, the regulating measure will overshoot or undershoot. Hence, “*a delay in a feedback process is critical relative to rates of change in the system state that the feedback loop is trying to control*” (13). This means that the present AMR system map provides a generic picture of the overall situation. Therefore, the size of the effect and the errors would look different depending on the geographical region.

Taken all the pros and cons together, the model cannot be used to predict a quantitative effect of interventions. The model did not include the risk factors on an individual level, because the focus of the model was to describe overall drivers at population level/regulatory level. However, the benefit is that the model can be used to predict the relative effect of different interventions. Nevertheless, systems thinking is considered as a complementing approach to the statistical or reductionist (analytic) thinking (12). The global crisis of AMR endangers the realization of the SDGs (23). Thus, comprehensive studies that assess the AMR system need to be conducted with the subject of sustainable development. This would enable other systems that are interconnected to the AMR system, such as the economic, health, and biodiversity system, also to change. Nevertheless, further research is needed to evaluate the effects of using deep leverage points to conduct interventions.

## Conclusions

In this study, we were able to apply the systems thinking approach to create a conceptual model of how AMR occurrence is influenced by different factors. The model improved our understanding of interconnections and feedbacks between system elements for the establishment of effective interventions. Regardless of some methodological drawbacks, feedback loops depict the potential to act flexible on changes, integrate new knowledge continuously, and adapt the system map to local circumstances. However, our model of the present AMR system map does not show how to solve the AMR challenge, but encourage scientists to deviate only from a linear thinking.

Overall, the systems thinking approach include different levels (i.e., micro, meso, and macro), which gives the study a holistic perspective and can be related to real-life scenarios. Due to the large number of stakeholders involved in the AMR network and bacteria's biological characteristics (i.e., spread between humans, animals, and environment), determining interventions need to be re-thought and require a circular approach. We suggest that this can contribute to more efficient interdisciplinary cooperation (i.e., one health), because the system map depicts different fields in one model.

This study shows that scientific approaches construct and bound our understanding of where we can intervene in systems. In the scientific field, we still sit broadly in the paradigm of “problem is on par with solution.” However, the system approach cannot stand alone but must be understood as an extension of the linear cause and effect research methods to improve the validity of results. Therefore, we suggest further research that applies interdisciplinary approaches (i.e., sustainability science) and addresses this global health issue with an intent of a system wide transformative change. Eventually, we conclude that the scope of systems thinking can beneficially connect AMR's overlapping scientific fields and aid in pursuing a common goal to fulfill the 2030 Agenda for Sustainable Development.

## Data Availability Statement

The original contributions presented in the study are included in the article/Supplementary Material, further inquiries can be directed to the corresponding author.

## Author Contributions

LM and HV conceived the idea to integrate socioeconomic factors with AMR abundance in a system map, elaborated the idea, and planned the study. LM conducted the literature research and generated the systems maps with assistance of HV. TH supported the work integrating the One Health approach and reviewing the conceptualized feedback loops. LM wrote the first draft. All authors have seen and accepted the final version.

## Conflict of Interest

The authors declare that the research was conducted in the absence of any commercial or financial relationships that could be construed as a potential conflict of interest.

## Publisher's Note

All claims expressed in this article are solely those of the authors and do not necessarily represent those of their affiliated organizations, or those of the publisher, the editors and the reviewers. Any product that may be evaluated in this article, or claim that may be made by its manufacturer, is not guaranteed or endorsed by the publisher.
