# Supplementary material for: System Mapping of Antimicrobial Resistance to Combat a Rising Global Health Crisis
Source: Front Public Health. 2022 Jun 17;10:816943. doi: 10.3389/fpubh.2022.816943 (PMC9249020; doi:10.3389/fpubh.2022.816943)
Supplement: Supplementary file 1 [file Data_Sheet_1.PDF]

System mapping of antimicrobial resistance to combat a rising global health crisis

Supplementary Materials

Supplementary tab. 1: Key terms of the Systems Thinking

**Word**	Definition	Source
**Backcasting**	*"It is a method in which the future desired conditions are envisioned, and steps are then defined to attain those conditions, rather than to take steps that are merely a continuum of present methods extrapolated into the future."*	Holmberg & Robert (2000)
**Emergence**	*"Properties of the system as a whole rather than properties that can be derived from the properties of the system components. Emergent properties are a consequence of the relationships among system components."*	Monat & Gannon (2015)
**Events**	*"Things that happen that we can see or observe."*	Monat & Gannon (2015)
**Feedback Loops**	*"It is the consistent behavior pattern over a long period of time that is the first hint of the existence of a feedback loop. […] A feedback loop is formed when changes in a stock affect the flows into or out of that same stock."*	Meadows (2008)
**Negative (-)/ Balancing Loops**	*"[…] balancing loops are generally stabilizing or goal seeking." "Balancing loops are continually trying to keep a system at some desired level of performance […]"*	Kim (1999)
**Positive (+)/ Reinforcing Loops**	*"[…] reinforcing loops destabilizes systems (that is, puts them out of equilibrium".*	Kim (1999)
**Leverage Points**	*"These are places within a complex system (a corporation, an economy, a living body, a city, an ecosystem) where a small shift in one thing can produce big chances in everything."*	Meadows (1999)
**Mental Models**	*"Paradigms or belief structures that attempt to interpret and/or simplify the universe in which we live."*	Monat & Gannon (2015)
**Patterns**	*"Sets of consistent and recurring observable events. Patterns may be physical, behavioral, or mental. Patterns are usually caused by underlying systemic structures and forces."*	Monat & Gannon (2015)
**Self-organization**	*"The tendency of a system to develop structures or patterns without the intervention of a designer or central plan, simply because of the interactions among the system elements."*	Monat & Gannon (2015)
**System Structures**	*"The manner in which a system's elements are organized or interrelated."*	Monat & Gannon (2015)
**Unintended Consequences**	*"Results of actions that were nether planned nor foreseen due to a lack of systems thinking."*	Monat & Gannon (2015)

Supplementary tab. 2: Overview of literature research

Database	Keywords	Results
**Topic: Problem Analysis AMR**		
	Timeline of research: **August**-September 2020<br>Filter: Not older than 5 years: 2020-2015<br>First 10 results were viewed	
**Scopus**	amr AND global AND surveillance	146
	amr AND "global surveillance"	11
	amr AND "socio- economic factor"	5
	amr AND "educational attainment"	345
	amr AND "infection and malnutrition"	1
	amr AND "cultural Traditions"	62
	amr AND review	700
	amr AND review AND intervention	106
	amr AND review AND policy	99
	amr AND review AND wicked problem	2
	amr AND "one health" AND agriculture	24
**Google Scholar**	amr AND "socio-economic factor"	44
	amr AND "one health"	>1,000
	antimicrobial resistance surveillance one health	>1,000
	antimicrobial resistance AND "one health" AND gene	>1,000
	amr AND review AND "wicked problem"	>1,000
**Topic: AMR and System Map**		
	Timeline of research: **September**-October 2020	
**Scopus**	Backcasting AND AMR	0
	Backcasting AND "One Health"	0
	"One Health" AND "system change"	2
	Backcasting AND system change	27
	"System map" AND "wicked problem"	19
	"System change" AND "wicked problem"	10
	"wicked problem" AND "antimicrobial resistance"	6
	"system map" AND "climate change"	17
	"system map" AND "solution"	64
	"system map*" AND "antimicrobial resistance"	5
	"system dynamic" AND "antimicrobial resistance"	0
	"system change" AND "antimicrobial resistance"	4
	"system approach" AND "antimicrobial resistance" OR AMR	3
**Google Scholar**	"system map*" AND "antimicrobial resistance"	51
	"system dynamic" AND "antimicrobial resistance"	45
	"system change" AND "antimicrobial resistance"	489
	"systems thinking applied to antimicrobial resistance"	>1,000

Supplementary tab. 3: List of linkage between system elements and literature research. Decisions to incorporate system elements into present AMR system map.

Number	System or System Element	Type	Linkage: (A) Literature search / (B) Henriksen et. al (2019)	Relevant sources
**1**	**Educational system**	Outside	**A:** Knowledge was linked to AMR<br>**B:** Education potentially associated with AMR abundance	Global Action Plan (GAP): WHO (2015)
**2**	**Wastewater treatment**		**A:** Not discussed<br>**B:** Untreated Sewage	Cantas et al. (2013), p. 7:<br>"*Wastewater treatment plants reduce the load of AMR bacteria, but treated water still carries elevated levels of AMR bacteria, and may select for strains with high levels of multidrug-resistance*".
**3**	**Transportation network (travel)**		**A:** Spread of resistant bacteria streams through travel and trade activities of animals and humans.<br>**B:** No approval of linkage	GAP: WHO (2015)<br>Cantas et al. (2013)<br>Pagani et al. (2020)
**4**	**Social structure**	Internal	**A:** Growing population. Africa deficient infrastructure.<br>**B:** Diverse AMR abundance among continents.	UN (2019)<br>Foster & Briceno-Garmendia (2010)
**5**	**Food demand/ security**		**A:** Food system contributes to the expansion of AMR worldwide.	FAO (2020)<br>WHO (2019)
**6**	**Antimicrobial usage**		**A:** Antimicrobials are present in the environment as a consequence of usage.	GAP: WHO (2015)<br>Pagani et al. (2020)<br>Pollock et al. (2020)
**7**	**(Intensified) Animal production**		**A:** Infection rates of animals related to industrialization causing AMU.	FAO (2020)<br>Kahn (2017)<br>O'Neill (2015)<br>Samanta et al. (2020)
**8**	**Collaboration partners**		**A:** AMS.<br>Top-down and bottom-up approach.<br>Wide stakeholder network.	Hermsen et al. (2020)<br>Birgand et al. (2018)<br>GAP: WHO (2015)<br>ReAct Europe (2016)
**9**	**Affluence**		**A:** Wealth.<br>Gap of knowledge due to missing research in LMICs.<br>Rising inequality and poverty in developing countries.<br>**B:** HDI was associated with AMR abundance.	Aarestrup & Woolhouse (2020)<br>UN (2020)
**10**	**Death/Year**		**A:** Increasing mortality.<br>**B:** Female Child Mortality Rate was associated on AMR abundance.	Chatterjee et al. (2018)<br>Kahn (2017)<br>Anderson, Schulze, et al. (2019)
**11**	**Antimicrobial resistance**		**A:** AMR interlinked with multiple and unknown factors<br>**B:** Association with several socio-economic factors.	GAP: WHO (2015)<br>WHO (2020)<br>IACG (2019)<br>Pagani et al. (2020)<br>Chatterjee et al. 2018
**12**	**Biodiversity**		**A:** Interlinkage to other systems (environments). Side effect of intensified agriculture. Beneficial to empower individuals and enhance resilience towards other crisis (i.e., hunger, climate change)	Erisman et al. (2016)<br>Landis (2017)

**13**	**Available resources**		**A:** Access to medicine. Water, Sanitation and Hygiene (WASH). Education<br>**B:** 18 associating socio-economic variables.	GAP: WHO (2015)<br>WHO (2020)
**14**	**Malnutrition/ infections**		**A:** Lack of WASH. Antibiotics for treatment. SDGs.<br>**B:** Association with AMR abundance	WHO (2020)<br>Alividza et al. (2018)<br>UN (2021)
**15**	**GNI per capita**		**A:** Economic system	GAP: WHO (2015)
**16**	**Natural environment**	External	**A:** One health, transmission of bacteria through e.g., water source.	IACG (2019)<br>Pagani et al. (2020)<br>Chatterjee et al. 2018
**17**	**Treatment method**		**A:** The transmission of infections in hospital settings is conditioned by the control of hygiene and infection prevention.<br>Culture may influence medicine intake.	WHO (2020)<br>GAP: WHO (2015)<br>Abdullahi (2011)
**18**	**Policies**		**A:** Recognized a need for system transformation of AMR.	GAP: WHO (2015)<br>Cantas et al. (2013)<br>Jørgensen et al. (2017)<br>WHO (2020)<br>Hermsen et al. (2020)

Supplementary tab. 4: Results of the Leverage points that were identified in the AMR system, following Meadows (1999), division into deep and shallow leverages according to Abson et al. (2017). The right column lists the identified characteristics of the AMR system. Number 1-3 outlines the intent of a future AMR system and establish the underlying values and goals. Number 4-6 builds the design of the current system and the social structures and institutions that govern the parameters. Number 7-9 show the internal dynamics that drive the system. Number 10-12 demonstrate the parameters, typically targeted by policy makers, which rule the mechanism. The interventions that would be powerful to transform the system was to empower the societal behavior of individuals that maintain low AMR levels (4) and to create educational awareness (2). Furthermore, to lower the risk of infection and malnutrition through equal access of clean water and sanitation to everyone (4) and to increase the careful handling of antimicrobials through advancements of the information flow (6).

**Order**	**System Charac-teristics**	**Places to Intervene**	**AMR System**
**12**		Constants, parameters, numbers	The conditions of living for animals as for humans contribute to the risk of infection, treated with antimicrobials (Alividiza et al., 2018; Samanta et al., 2020). Worldwide 700,000 people die yearly due to AMR, with an increasing trend (Kahn, 2017). Economic loss through longer treatment periods and higher prices of valid antibiotics (health system) and productivity loss of ill people.
**11**	Parameters	The sizes of buffers and other stabilizing stocks, relative to their flows	There is uncertainty about the buffers or rather the sustained efficiency of antibiotics in the long term. It depends on the type of antibiotic, the geographical location and individual health profile (Henriksen et al., 2019; Alividza et al., 2018). Integrated approaches with several stakeholders involved are suggested to stabilize stocks in the long term and potentially decrease AMU (Boriani et al., 2017).
**10**		The structure of materials stocks and flows	Antimicrobials circulate between people, animals and the environment (Littman et al., 2020). People that have access to the health system also have access to antibiotics (i.e., AMU). They will also have possible access to vaccines, which can prevent illness in the first place (Jansen et al., 2018). Animals from intensified agriculture are exposed to antibiotics and working people at farms (Samanta et al., 2020; O'Neill, 2015; ReAct Europe, 2016).
**09**		The lengths of delays, relative to the rate of system change	With an increase of AMU, AMR rises globally (Henriksen et al., 2019). However, it takes several years from AMR development to widespread dissemination with implications for treatment. The researchers are still assessing several determining pathways causing AMR, which are complex and encompass uncertainties (WHO, 2020).
**08**	Feedbacks	The strength of negative feedback loops relative to the impacts they are trying to correct against	Withhold the efficiency of antibiotics and avoid economic loss (WHO, 2015; IACG, 2019). Access to resources to decrease risk of infections and malnutrition (Alividza et al. 2018). Alternative treatment methods than antimicrobials avoid undesirable side effects of antibiotics (Jansen et al., 2018). Protect the environment against the spread of antimicrobials, enhance biodiversity instead of intensified agriculture or increase hygienic farming conditions in other ways (Erisman et al., 2016; Landis, 2017; Boriani et al., 2017). Increasing self-sufficiency of individuals (Abson et al., 2017; Meadows, 1999).
**07**		The gain around driving positive feedback loops	Treatment of acute infections with antibiotics to cure infections and potentially avoid deaths as well as ensuring peoples' workforce (WHO, 2020). The growth model of developed countries to increase the yield on short-term (Chatterjee et al., 2018).
**06**		The structure of information flows	Integrative approach between multiple stakeholders, including micro-, macro- and meso-level, and transparency along the food supply chain (Boriani et al., 2017). Increasing antimicrobial stewardship (Hermsen et al., 2020). Technological innovation, such as digital platform to share knowledge (Boriani et al., 2017).
**05**		The rules of the system	Rewriting the rules of the system. Reorganising the social structure of the low HDI countries to prevent poverty (i.e., infection and malnutrition) (Alividza et al., 2018). Prevention of food contamination and security by securing a food safety framework for farmers in low HDI countries and strengthen the income of farmers (Boriani et al., 2017 Pagani et al., 2020).
**04**	Design	The power to add, change, evolve, or self-organize system structure	Sanitation facilities and access to water raise the hygienic circumstances to prevent the risk of infections and malnutrition for animals and humans (WHO, 2015). Integrating producing farmer community and their operations properly along the food chain (i.e., interacting with food industry and consumers) (Boriani et al., 2017).
**03**	Intent	The goals of the system	Maintain the effectiveness of antimicrobials and protect the treatment of life-threatening diseases (WHO, 2015; IACG, 2019; FAO, 2020; Kahn, 2017; Alividza et al., 2018; Chatterjee et al., 2018; Samanta et al., 2020; Pagani et al., 2020; Littman et al., 2020).
**02**		The mindset or paradigm out of which the system arises	Creating educational awareness (Hermsen et al, 2020).
**01**		The power to transcend paradigms	Empowerment of individuals through resources to gain access to water, sanitation and health (WHO, 2020; WHO 2015; WHO 2019).

Shallow leverage points (orders 12–08)

Deep leverage points (orders 08–01)
